# Circulating Tumor DNA as a Potential Marker to Detect Minimal Residual Disease and Predict Recurrence in Pancreatic Cancer

**DOI:** 10.3389/fonc.2020.01220

**Published:** 2020-07-30

**Authors:** Jiahong Jiang, Song Ye, Yaping Xu, Lianpeng Chang, Xiaoge Hu, Guoqing Ru, Yang Guo, Xin Yi, Liu Yang, Dongsheng Huang

**Affiliations:** ^1^Key Laboratory of Tumor Molecular Diagnosis and Individualized Medicine of Zhejiang Province, Department of Medical Oncology, Zhejiang Provincial People's Hospital, People's Hospital of Hangzhou Medical College, Hangzhou, China; ^2^Division of Hepatobiliary and Pancreatic Surgery, Department of Surgery, The Second Affiliated Hospital, School of Medicine, Zhejiang University, Hangzhou, China; ^3^Department of Translational Medicine, Geneplus-Beijing Institute, Beijing, China; ^4^Department of Pathology, Zhejiang Provincial People's Hospital, People's Hospital of Hangzhou Medical College, Hangzhou, China

**Keywords:** pancreatic cancer, minimal residual disease, circulating tumor DNA, disease recurrence, KRAS

## Abstract

Pancreatic ductal adenocarcinoma (PDAC) is one of the leading causes of cancer death, partly due to the high recurrence rates for patients with PDAC. Current postoperative surveillance methods, including monitoring of clinical symptoms, tumor markers, and CT imaging, lack sensitivity and specificity for minimal residual disease (MRD). We investigated whether the detection of circulating tumor DNA (ctDNA) could identify MRD and predict relapse in postoperative patients with PDAC. In this study, we performed panel-captured sequencing to detect somatic mutations. Matched tissue samples were obtained to verify mutation. A total of 27 patients and 65 plasma samples were included. Among the somatic mutations, KRAS and TP53 were the most recurrent genes in both tissue and plasma samples. The detectable rate of ctDNA increased with the stage of PDAC. The maximal variant allele fraction (VAF) of ctDNA had a positive correlation with tumor largest diameter (*p* = 0.0101). Patients with ctDNA-positive status postoperatively had a markedly reduced disease-free survival (DFS) compared to those with ctDNA-negative status (HR, 5.20; *p* = 0.019). Positive vascular invasion significantly influenced disease-free survival (DFS) (*p* = 0.036), and positive postoperative ctDNA status was an independent prognostic factor for DFS (HR = 3.60; 95% CI, 1.15–11.28; *p* = 0.028). Postoperative ctDNA detection provides strong evidence of MRD and identifies patients with a high risk of relapse. ctDNA detection is a promising approach for personalized patient management during postoperative follow-up.

## Introduction

Pancreatic ductal adenocarcinoma (PDAC) is the seventh leading cause of cancer deaths in China, with ~90.1 new cancer cases and 79.4 cancer deaths (per 100,000) projected to occur every year (1). Surgical resection is a potentially curative treatment that could improve the overall 5-years survival rate from 8 to 25% in PDAC (2, 3). Unfortunately, disease recurrence severely influences the outcomes of postoperative patients with PDAC. The current criterion to evaluate disease recurrence is based on the serum tumor marker cancer antigen 19-9 (CA19-9) and computed tomography. However, the standard biomarker CA19-9 has limited sensitivity and specificity in PDAC and is even negative in Lewis (-) individuals (4). Only macroscopic disease recurrence can be detected through CT surveillance, and identification is usually uncertain due to normal tissue changes after surgery. Recurrence risk was associated with some clinical and pathological features, such as poorly differentiated histology, maximum tumor size, and positive lymph node status. Nevertheless, only these factors were inadequate to assess recurrence risk accurately (5). Indeed, more than 70% of postoperative patients with PDAC will die from recurrent disease (6); thus, a reliable approach is urgently needed to identify minimal residual disease (MRD) and predict the recurrence risk for PDAC.

Liquid biopsy is a promising approach for disease surveillance in solid tumors (7). Emerging evidence has shown that circulating tumor DNA (ctDNA) analysis can identify MRD shortly after surgery in patients with non-metastatic colon cancer and breast cancer (8, 9). These studies indicated that ctDNA detection can predict cancer recurrence with high sensitivity. Meanwhile, ctDNA detection is a promising approach for personalized patient management during postoperative follow-up due to its non-invasive, real-time, and dynamic features.

In this study, we aimed to determine whether ctDNA analysis can reliably identify MRD in postoperative patients with PDAC and compare dynamic changes in ctDNA with ordinary tumor surveillance during treatment.

## Materials and Methods

### Patients and Samples

Between July 2016 and September 2018, a total of 27 patients diagnosed with PDAC were enrolled at Zhejiang Provincial People's Hospital. Plasma samples were collected at these time nodes: preoperation, postoperation (7-days after surgery), and each follow-up visits (1 or 3 months after operation) (Table S1). Surgical tumor tissue samples were obtained from formalin-fixed paraffin-embedded tissues. According to the Response Evaluation Criteria in Solid Tumors (RECIST) version 1.1, a computed tomography scan and cancer antigen 19-9 (CA 19-9) are used to assess treatment effectiveness and monitor tumor progression every 1–3 months. This study was approved by the ethical committee at Zhejiang Provincial People's Hospital (No. 2016KY129). All participants provided written informed consent before any study-related operation was performed.

### Genomic DNA Extraction

In each eligible patient, at least 10 ml of peripheral blood was collected to isolate plasma and lymphocytes. All samples were stored at −80°C prior to DNA extraction. QIAamp DNA Blood Mini Kits and QIAamp DNA Mini Kits (Qiagen, Hilden, Germany) were used to extract genomic DNA from plasma lymphocytes (germline DNA) and tumor tissue (tumor DNA), respectively. Circulating cell-free DNA (cfDNA) was extracted from plasma using the QIAamp Circulating Nucleic Acid Kit (Qiagen, Hilden, Germany). cfDNA released by tumor cells was termed as ctDNA, which carries tumor-specific genetic mutations, including somatic single nucleotide variations (SNVs) and somatic insertions/deletions (Indels). DNA concentrations were quantified using a Qubit fluorometer (Invitrogen, Carlsbad, CA, USA). The length of cfDNA fragments was assessed using the Agilent 2100 BioAnalyzer (Agilent Technologies, Santa Clara, CA, USA).

### Library Preparation, Hybridization Capture, and Sequencing

Germline and tumor DNA was fragmented into 200–250 bp segments using a Covaris S2 instrument (Woburn, MA, USA). After an end repair and A-tailing reaction, adapters with unique base sequences (unique identifiers, UIDs) were ligated to both ends of the double-stranded molecules, and then fragment amplification was performed using PCR. Indexed Illumina NGS libraries were prepared for germline DNA, tumor DNA, and cfDNA using the NEB DNA Library Preparation Kit (NEB, MA, USA). Subsequently, constructed libraries were hybridized to custom-designed biotinylated oligonucleotide probes (IDT, Coralville, IA, USA) covering 1,017 cancer susceptibility genes (Table S2). DNA sequencing was performed on a HiSeq2000 System (Illumina, CA, USA). The sequencing protocol was executed as previously described (10).

### Raw Data Processing

Adapter sequences from the raw data and reads with a high *N* rate (>50%) or low-quality bases (>50%, Q < 5) were filtered out to obtain clean data. The clean reads were aligned to the hg19 human genome using the Burrows-Wheel Aligner (BWA, http://bio-bwa.sourceforge.net/) program. Subsequently, duplicate reads were identified using Picard's Mark Duplicates tool (https://software.broadinstitute.org/gatk/documentation/tooldocs/4.0.3.0/picard_sam_markduplicates_MarkDuplicates.php). Base quality recalibration and local realignment were performed using The Gene Analysis Toolkit (GATK, https://www.broadinstitute.org/gatk/).

### Mutation Identification

SNVs and somatic Indels were identified using GATK and MuTect2 (https://software.broadinstitute.org/gatk/documentation/tooldocs/3.8-0/org_broadinstitute_gatk_tools_walkers_cancer_m2_MuTect2.php) and filtered by the sequencing results of peripheral blood lymphocytes. All variants underwent further filtration with the following criteria: 1) variants with <5 high-quality reads were removed (mapping quality ≥ 30, base quality ≥ 30); 2) variants included in the false positive database were removed; 3) variants with <0.1% mutant frequency that were included in several single nucleotide polymorphism databases (dbsnp, https://www.ncbi.nlm.nih.gov/projects/SNP/; 1000G, https://www.1000genomes.org/; ESP6500, https://evs.gs.washington.edu/; ExAC, http://exac.broadinstitute.org/) were retained; and 4) exonic or splicing variants were retained while synonymous variants were removed. The retained variants following this filtration were denoted as high-confidence somatic variants.

### Statistical Analysis

The relationship of maximal variant allele fraction (VAF), CEA, CA19-9 and tumor largest diameter (TLD) was assessed using linear analysis. Categorical time-to-event analyses of disease-free survival were conducted using the Kaplan–Meier method with log-rank test to estimate *p*-values, and the Cox exp (beta) method was used to estimate hazard ratios. Univariate and Multivariate Cox analyses for risk factors for relapse were performed using SPSS 22.0 (IBM, Armonk, NY, USA). A *p* < 0.05 was considered significant.

## Results

### Patient Characteristics

In this study, we profiled 65 plasma and 27 tissue samples from 27 eligible patients with PDAC. The clinical characteristics of all patients are summarized in Table 1. All patients were treated with surgical resection. As of 14 November 2018, the median follow-up was 18.6 months (range 12.4–28.9 months), and 14 patients experienced relapse.

**Table 1:** Clinical characteristics of enrolled patients.

**Characteristics**	**Total (*n* = 27)**
**Age (years)**	
Median (range)	62 (43–82)
**Sex, no. (%)**	
Male	17 (62.97)
Female	10 (37.04)
**Stage, no. (%)**	
I	13 (48.15)
II	9 (33.34)
IV	5 (18.52)
**Lymph nodes metastasis, no. (%)**	
Positive	7 (25.93)
Negative	20 (74.07)
**Nerve invasion, no. (%)**	
Positive	17 (62.96)
Negative	10 (37.04)
**Vascular invasion, no. (%)**	
Positive	10 (37.04)
Negative	17 (62.96)
**Differentiation, no. (%)**	
Moderately-poor	15 (55.56)
High	12 (44.44)
**Tumor size, no. (%)**	
>4 cm	12 (44.44)
≤4 cm	15 (55.56)

ctDNA was successfully extracted from all plasma samples with an average concentration of 28.38 ng/ml (range, 5.57–119.10 ng/ml). The average coverage depths for sequenced tumor DNA and ctDNA were 864.64 × (range, 371×-1590.80×) and 1323.18× (range, 719.92×-2423×), respectively, and the fractions of target region coverage were all above 99%.

### Mutant Prevalence of Plasma and Tissue Samples

**Figure 1:**
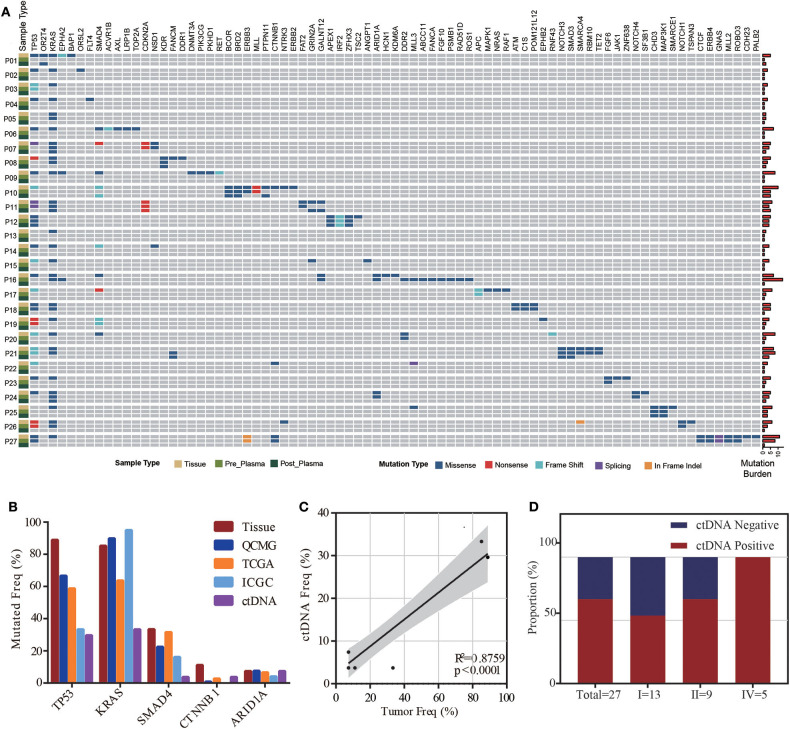
ctDNA and tissue mutational landscape in patients with pancreatic ductal adenocarcinoma (PDAC). **(A)** Heatmap illustrating tissue, preoperative and postoperative ctDNA mutations identified in this study. Right bars indicate the mutation burden of each sample. **(B)** Comparison of mutation frequencies in our study (tissue and preoperative ctDNA) and public tissue databases (QCMG, TCGA, and ICGC). **(C)** Correlation between mutation rates in preoperative ctDNA and tumor DNA. **(D)** The detectable rate of different clinical stages in preoperative plasma samples from 27 PDAC patients.

The mutant prevalence of tissue samples and plasma samples collected before surgery is shown in Figure 1A. In total, 153 somatic mutations were identified in tissue samples from 27 patients, including 123 missense mutations (80.39%), 8 non-sense mutations (5.23%), 16 frameshift mutations (10.46%), 1 deletion of a small fragment (0.65%), and 1 insertion of a small fragment (0.65%) in the coding sequence. In addition, we also identified one mutation in 3' splice sites (0.65%) and three mutations in 5' splice sites (1.96%). TP53 (24/27, 88.89%), KRAS (23/27, 85.19%), and SMAD4 (9/27, 33.34%) were the most frequent mutant genes in the tissue samples. Interestingly, co-mutants TP53/KRAS, TP53/SMAD4, and KRAS/SMAD4 occurred in 20 (74.04%), 9 (33.34%), and 7 patients (25.93%), respectively. Mutant TP53, KRAS, and SMAD4 were co-expressed in seven patients (25.93%). In contrast, ctDNA was detected in 18 of 27 preoperative plasma samples including 65 somatic mutations. Frequencies of KRAS and TP53 reached 50.00% (9/18) and 44.45% (8/18) in preoperative ctDNA positive patients, respectively. Mutant KRAS and TP53 co-occurred in four patients (14.81%). To confirm the validity of the sequencing results, we compared the prevalence of mutations detected in our analysis to those detected in publicly available PDAC tissue datasets (QCMG, TCGA, and ICGC) (11–13). The top 5 mutant genes in our study and public datasets were listed in Figure 1B. The gene mutation rates of tumor tissue in our cohort were generally higher than those in public datasets except KRAS, possibly due to the low depth of whole exon sequencing and racial difference. We also assessed the correlation between the prevalence of mutations in tumor DNA and ctDNA in our cohort. The mutation rates of the top 10 genes in ctDNA were significantly correlated with those in tumor DNA (*R*^2^ = 0.875; *p* < 0.0001) (Figure 1C). There were also some differences between tumor DNA and ctDNA, such as the lower frequency of TP53 in ctDNA (Figure 1B).

### ctDNA Detection and Clinical Feature Analysis

**Figure 2:**
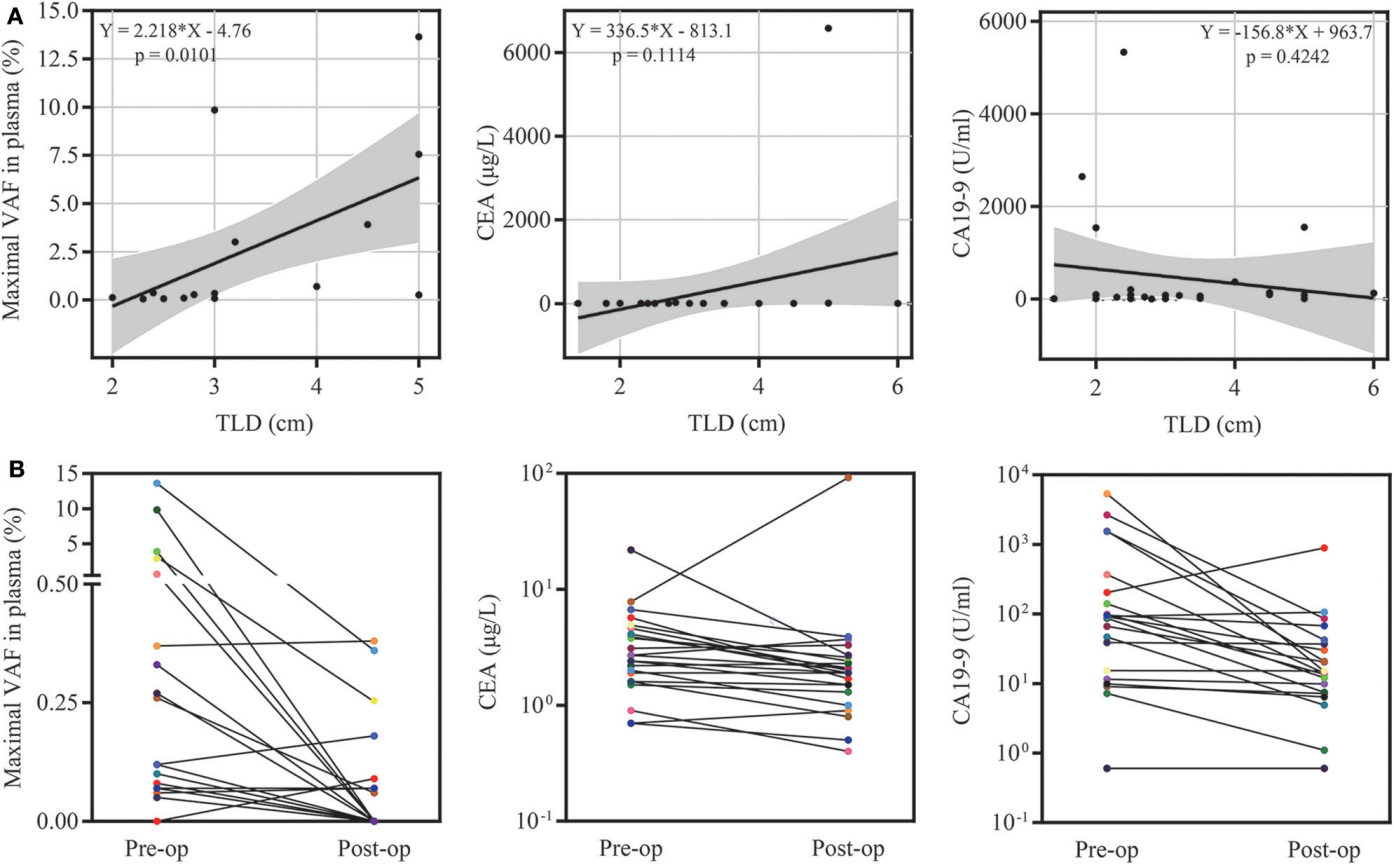
ctDNA burden and clinical feature analysis. **(A)** The relationship of the maximal variant allele fraction (VAF), CEA, CA19-9 and tumor largest diameter (TLD). **(B)** Dynamic changes in the maximal VAF, CEA and CA19-9 7 days after surgery.

ctDNA was detected in 18 of 27 preoperative plasma samples, resulting in a detectable rate of 66.67%. The majority of patients were stage I (*n* = 13, 48.15%) and stage II (*n* = 9, 33.34%), while only five patients were stage IV (*n* = 5, 18.52%) in this cohort. The detectable rate increased with the stage of PDAC (from 53.8 to 66.7%, reaching 100% for stage IV) (Figure 1D). To determine whether the ctDNA burden is associated with tumor size, the maximal VAF of 18 ctDNA detectable patients, CEA and CA19-9 were assessed preoperatively to identify the correlation between TLDs. Linear analysis revealed a positive correlation between the maximal VAF (ranged from 0.05 to 13.64%) in plasma and the TLD (*p* = 0.0101), while CEA and CA19-9 showed no distinct relevance (*p* = 0.1114, *p* = 0.4242) (Figure 2A). After 7 days of surgical resection, the status of ctDNA was changed in 19 patients, in which one turned positive and 10 turned negative completely. We compared the changes of maximal VAF between preoperative (ranged from 0.00 to 13.64%) and postoperative (ranged from 0.00 to 0.38%) plasma, the maximal VAF level was significantly decreased when compared to the preoperative levels in most patients (*p* = 0.036). However, only slight changes appeared in CEA (*p* = 0.346) and CA19-9 levels (*p* = 0.196), suggesting a better sensitivity of ctDNA for detecting dynamic tumor changes (Figure 2B).

### Landmark Analysis of Prognosis for Postoperative Patients

**Figure 3:**
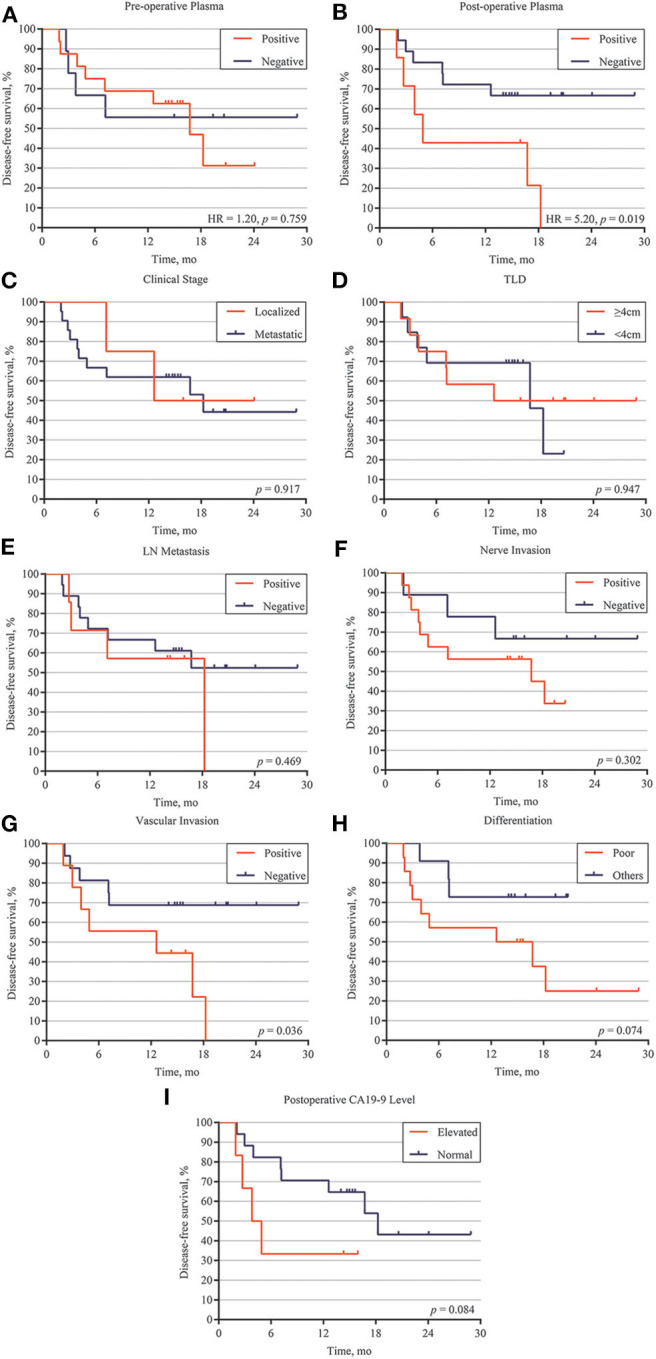
Correlation between ctDNA, clinical high-risk factors and disease relapse. **(A)** Disease-free survival analysis of preoperative ctDNA detection patients with PDAC. **(B)** Disease-free survival analysis of postoperative ctDNA detection patients with PDAC. Disease-free survival analysis of 27 patients with different clinical features, including localized /metastatic clinical stage **(C)**, tumor largest size **(D)**, lymph node (LM) metastasis **(E)**, nerve invasion **(F)**, vascular invasion **(G)**, differentiation **(H)**, and postoperative CA19-9 level **(I)**.

To investigate serial ctDNA analysis for disease surveillance during follow-up, we performed postoperative monitoring of 27 patients with ctDNA analysis, tumor biomarkers, and CT scans. Postoperative ctDNA was positive in nine patients, and eight of these patients ultimately recurred. Patients with ctDNA-positive status postoperatively had a markedly reduced disease-free survival (DFS) compared to those with ctDNA-negative status (HR, 5.20; *p* = 0.019) (Figure 3B). However, preoperative ctDNA status showed no significant effect on DFS (HR, 1.20; *p* = 0.759) (Figure 3A).

We also explored the correlation between DFS and clinical risk factors to help predict patient prognosis. Survival analysis demonstrated that localized or metastatic clinical stage had no difference on patient DFS (*p* = 0.917) (Figure 3C). Other risk factors, including larger tumor size (>4 cm), positive lymph node metastasis, positive nerve invasion, moderate-poor differentiation, and postoperative CA19-9 level, also showed the same results (*p* > 0.05) (Figures 3D–F,H,I). However, patients with positive vascular invasion had significantly lower freedom from progression than those without vascular invasion (*p* = 0.036) (Figure 3G). To further validate the correlation of postoperative ctDNA status and disease recurrence, we performed a Cox analysis for the aforementioned risk factors and postoperative ctDNA status. The results of the univariate analysis were consistent with those of the survival analysis, in which only vascular invasion (HR, 3.16; 95% CI, 0.93–10.79; *p* = 0.036) and postoperative ctDNA status (HR, 3.55; 95% CI, 0.90–13.89; *p* = 0.019) were associated with disease relapse. Several risk factors, such as differentiation, nerve invasion, and postoperative CA19-9 levels, also showed a tendency to affect disease recurrence but failed to obtain positive results due to the small sample size. Subsequently, multivariate analysis was performed to adjust for the potential effects of differentiation, lymph node metastasis, nerve invasion, and vascular invasion. With these adjustments, postoperative ctDNA detection still affected disease recurrence in patients with PDAC (HR, 3.60; 95% CI, 1.15–11.28; *p* = 0.028) (Table 2).

**Table 2:** Univariate and multivariate cox analysis for risk factors of relapse.

**Variable**	**Univariate analysis**		**Multivariate analysis**	
	**HR (95% CI)**	***p***	**HR (95% CI)**	***p***
Clinical stage, Metastatic vs. Localized	0.92 (0.21–4.05)	0.917	-	-
Tumor size, ≥4 vs. <4 cm	0.96 (0.31–2.99)	0.947	-	-
Differentiation, Poor vs. Other	3.08 (0.99–9.56)	0.074	2.30 (0.51–10.45)	0.279
Lymph node metastasis, Positive vs. Negative	1.55 (0.41–5.77)	0.469	1.14 (0.29–4.50)	0.850
Nerve invasion, Positive vs. Negative	1.96 (0.62–6.24)	0.302	1.51 (0.35–6.43)	0.578
Vascular invasion, Positive vs. Negative	3.16 (0.93–10.79)	0.036^*^	1.50 (0.23–9.82)	0.673
Postoperative CA19-9 level, Elevated vs. Normal	2.69 (0.57–12.75)	0.084	-	-
Preoperative ctDNA status, Positive vs. Negative	0.65 (0.20–2.05)	0.453		
Postoperative ctDNA status, Positive vs. Negative	3.55 (0.90–13.89)	0.019^*^	3.60 (1.15–11.28)	0.028^*^^a^

* *Statistical significance*.

a *Multivariate analysis was performed to adjust for the potential effects of differentiation, lymph node metastasis, nerve invasion, and vascular invasion*.

## Discussion

Pancreatic ductal adenocarcinoma is one of the most lethal diseases and has a poor prognosis, which may be correlated with disease recurrence and the lack of effective monitoring methods. Recently, several studies have demonstrated that ctDNA profiling is associated with tumor burden and can predict tumor recurrence in patients with colon cancer and breast cancer (8, 9); however, research that focuses on PDAC and NGS based ctDNA analysis is still scarce. We collected tumor tissue and blood specimens from 27 PDAC patients and performed panel-based NGS of all samples. We then evaluated the concordance between our plasma samples, matched tumor samples and public tissue datasets. The correlation of MRD, ctDNA status, and risk factors for relapse will also be discussed.

In our study, most mutant genes in tumor DNA showed higher mutation frequencies than those reported in public datasets, which may be partly due to low depth of whole exon sequencing or racial differences in public datasets. Notably, mutant genes detected in ctDNA had a lower frequency than those detected in matched tumor tissue. Given the biological features such as abundant extracellular matrix, the shedding into peripheral blood is usually poor for ctDNA in PDAC patients. Besides, PDAC is genomic heterogeneous and different mutations in the same tumor may yield distinct allele frequencies (AFs), and those with relatively low AFs may not yet reach limit of detection and thus are deemed as undetected. Thus, it's difficult to reflect the entire mutational landscape via ctDNA profiling for individual PDAC patient. However, we revealed the significant correlation between the prevalence of mutation in tumor DNA and ctDNA based on cohort level, indicating that current platform is eligible to detected tumor-derived mutations in plasma ctDNA. Actually, several studies about the utility of ctDNA in MRD identification have been reported, and usually the presence of one or two tumor-derived mutations in plasma ctDNA is associated with post-operative survival (14, 15). In these cases, the mutational spectrum of tumor tissue provides prior knowledge for mutation tracing in ctDNA, and the circulating tumor burden reflected by mutational AFs can serve as an indicator for tumor surveillance. The most frequently mutated genes were TP53 and KRAS in both the tissue and plasma samples, and these genes play an important role in tumor proliferation and recurrence (16, 17).

After further filtration, at least one tumor-specific mutation was detected in ctDNA from 27 patients, and nearly 60% of tumor-derived mutations were detected in matched ctDNA, indicating that ctDNA profiling is a reliable and non-invasive source of molecular characteristics for PDAC. A previous study demonstrated that the detectable rate of mutations in ctDNA was significantly increased in patients with late-stage tumors compared with those with early-stage tumors (18). In this study, the detectable rate of mutations in ctDNA increased with tumor stage and finally reached 100% for stage IV, which was consistent with previous findings.

CA19-9 is considered the most common biomarker in PDAC diagnosis, treatment monitoring, and survival prediction. However, the level of CA19-9 is also increased in many benign conditions, such as biliary disease, liver disease, and pancreatitis, and only applicable in Lewis (+) patients (4, 19). Compared to CA19-9, CEA shows lower sensitivity and specificity in PDAC (20). In this condition, the postoperative ctDNA analysis revealed a positive relationship between the maximal VAF and dynamic tumor burden changes and proved to be efficient for detecting relapse compared to CA19-9 and CEA. Besides, consistent with other studies, ~90% recurrence patients were postoperative ctDNA-positive before the time of radiologic relapse, indicating that postoperative ctDNA analysis may be more sensitive than CT imaging in MRD identification (21, 22).

The presence of postoperative ctDNA appeared to be a prognostic factor for poor DFS and OS. Pietrasz et al. demonstrated that patients with postoperatively undetectable ctDNA had a longer DFS (17.6 vs. 4.6 months) and OS (32.2 vs. 19.3 months) than those with detectable ctDNA (14). Hadano et al. reported that patients with positive ctDNA had only half the median OS duration of those without detectable ctDNA (13.6 vs. 27.6 months) (15). Our work also reveals that ctDNA analysis indicates MRD after surgery and predicts recurrence in patients with PDAC. Additionally, survival analysis and univariate analysis demonstrated that only positive vascular invasion could affect DFS, while other clinical risk factors showed no significant results. The small sample size may have caused the negative results of some risk factors. The multivariate analysis provided further proof for the detection of postoperative ctDNA as an independent prognostic factor for patients with PDAC. Two key limitations of our study are the small cohort and the lack of follow-up blood sample. However, these two limitations may not influence the accuracy and sensitivity of ctDNA detection.

In conclusion, this study revealed the utility of ctDNA detection as a prognostic biomarker in patients with PDAC. Highly precise ctDNA detection has the potential to transform clinical practice via non-invasive monitoring of solid tumor malignancies and identification of MRD at earlier time points than standard clinical surveillance.

## Data Availability Statement

The datasets presented in this study can be found in online repositories. The names of the repository/repositories and accession number(s) can be found below: the National Center for Biotechnology Information (http://www.ncbi.nlm.nih.gov/) GenBank database as individual BioProjects PRJNA634169.

## Ethics Statement

The studies involving human participants were reviewed and approved by the Ethical Committee of Zhejiang Provincial People's Hospital. The patients/participants provided their written informed consent to participate in this study.

## Author Contributions

LY and DH contributed to the conception of the study. JJ, SY, and YX contributed to experimental technology and experimental design. LC, YG, and GR provided molecular biology experimental technical support. JJ, SY, and XH performed the data analysis. XY helped perform the analysis with constructive discussions and paper modification. JJ, LY, and DH wrote the manuscript. All authors contributed to the article and approved the submitted version.

## Conflict of Interest

YX, LC, and XY were employed by the company Geneplus-Beijing Institute. The remaining authors declare that the research was conducted in the absence of any commercial or financial relationships that could be construed as a potential conflict of interest.

## References

[1] Chen W Zheng R Baade PD Zhang S Zeng H Bray F Cancer statistics in China, 2015. CA Cancer J Clin. (2016) 66:115–32. 10.3322/caac.21338 26808342

[2] Bilimoria KY Bentrem DJ Ko CY Stewart AK Winchester DP Talamonti MS. National failure to operate on early stage pancreatic cancer. Ann Surg. (2007) 246:173–80. 10.1097/SLA.0b013e3180691579 17667493 PMC1933550

[3] Ryan DP Hong TS Bardeesy N Pancreatic adenocarcinoma. N Eng J Med. (2014) 371:2140–1. 10.1056/NEJMra1404198 25427123

[4] Luo G Liu C Guo M Long J Liu Z Xiao Z. CA19-9-Low&Lewis (+) pancreatic cancer: a unique subtype. Cancer Lett. (2017) 385:46–50. 10.1016/j.canlet.2016.10.046 27840089

[5] Ghaneh P Kleeff J Halloran CM Raraty M Jackson R Melling J. The impact of positive resection margins on survival and recurrence following resection and adjuvant chemotherapy for pancreatic ductal adenocarcinoma. Ann Surg. (2019) 269:520–9. 10.1097/SLA.0000000000002557 29068800

[6] Spanknebel K Conlon KC. Advances in the surgical management of pancreatic cancer. Cancer J. (2001) 7:312–23. 11561607

[7] Diaz LA Jr Bardelli A. Liquid biopsies: genotyping circulating tumor DNA. J Clin Oncol. (2014) 32:579–86. 10.1200/JCO.2012.45.2011 24449238 PMC4820760

[8] Tie J Wang Y Tomasetti C Li L Springer S Kinde I. Circulating tumor DNA analysis detects minimal residual disease and predicts recurrence in patients with stage II colon cancer. Sci Transl Med. (2016) 8:346ra92. 10.1126/scitranslmed.aaf6219 27384348 PMC5346159

[9] Garcia-Murillas I Schiavon G Weigelt B Ng C Hrebien S Cutts RJ. Mutation tracking in circulating tumor DNA predicts relapse in early breast cancer. Sci Transl Med. (2015) 7:302ra133. 10.1126/scitranslmed.aab0021 26311728

[10] Yang L Wang G Zhao X Ye S Shen P Wang W. A novel WRN frameshift mutation identified by multiplex genetic testing in a family with multiple cases of cancer. PloS ONE. (2015) 10:e0133020. 10.1371/journal.pone.0133020 26241669 PMC4524609

[11] Bailey P Chang DK Nones K Johns AL Patch AM Gingras MC. Genomic analyses identify molecular subtypes of pancreatic cancer. Nature. (2016) 531:47–52. 10.1038/nature16965 26909576

[12] Waddell N Pajic M Patch AM Chang DK Kassahn KS Bailey P. Whole genomes redefine the mutational landscape of pancreatic cancer. Nature. (2015) 518:495–501. 10.1038/nature14169 25719666 PMC4523082

[13] Cancer Genome Atlas Research Network Electronic address, Cancer Genome Atlas Research N. Integrated genomic characterization of pancreatic ductal adenocarcinoma. Cancer Cell. (2017) 32:185–203.e13. 10.1016/j.ccell.2017.07.007 28810144 PMC5964983

[14] Pietrasz D Pecuchet N Garlan F Didelot A Dubreuil O Doat S. Plasma circulating tumor DNA in pancreatic cancer patients is a prognostic marker. Clin Cancer Res. (2017) 23:116–23. 10.1158/1078-0432.CCR-16-0806 27993964

[15] Hadano N Murakami Y Uemura K Hashimoto Y Kondo N Nakagawa N. Prognostic value of circulating tumour DNA in patients undergoing curative resection for pancreatic cancer. Br J Cancer. (2016) 115:59–65. 10.1038/bjc.2016.175 27280632 PMC4931379

[16] Kim H Park CY Lee JH Kim JC Cho CK Kim HJ. Ki-67 and p53 expression as a predictive marker for early postoperative recurrence in pancreatic head cancer. Ann Surg Treat Res. (2015) 88:200–7. 10.4174/astr.2015.88.4.200 25844354 PMC4384285

[17] Eser S Schnieke A Schneider G Saur D. Oncogenic KRAS signalling in pancreatic cancer. Br J Cancer. (2014) 111:817–22. 10.1038/bjc.2014.215 24755884 PMC4150259

[18] Bettegowda C Sausen M Leary RJ Kinde I Wang Y Agrawal N. Detection of circulating tumor DNA in early- and late-stage human malignancies. Sci Transl Med. (2014) 6:224ra24. 10.1126/scitranslmed.3007094 24553385 PMC4017867

[19] Poruk KE Gay DZ Brown K Mulvihill JD Boucher KM Scaife CL. The clinical utility of CA 19-9 in pancreatic adenocarcinoma: diagnostic and prognostic updates. Curr Mol Med. (2013) 13:340–51. 10.2174/1566524011313030003 23331006 PMC4419808

[20] Brody JR Witkiewicz AK Yeo CJ. The past, present, and future of biomarkers: a need for molecular beacons for the clinical management of pancreatic cancer. Adv Surg. (2011) 45:301–21. 10.1016/j.yasu.2011.04.002 21954696

[21] Scholer LV Reinert T Orntoft MW Kassentoft CG Arnadottir SS Vang S. Clinical implications of monitoring circulating tumor DNA in patients with colorectal cancer. Clin Cancer Res. (2017) 23:5437–45. 10.1158/1078-0432.CCR-17-0510 28600478

[22] Wang JSZ Sausen M Parpart-Li S Murphy DM Velculescu VE Wood LD Circulating tumor DNA (ctDNA) as a prognostic marker for recurrence in resected pancreas cancer. J Clin Oncol. (2015) 33:16971 10.1200/jco.2015.33.15_suppl.11025

