# Supplementary material for: Circulating Tumor DNA as a Potential Marker to Detect Minimal Residual Disease and Predict Recurrence in Pancreatic Cancer
Source: Front Oncol. 2020 Jul 30;10:1220. doi: 10.3389/fonc.2020.01220 (PMC7406781; doi:10.3389/fonc.2020.01220)
Supplement: Supplementary file 1 [file Data_Sheet_1.docx]

**Supplementary material**

| Table S1. Information of 65 plasma samples in 27 patients.   \| Case number \| Preoperation \| Postoperation^a^ \| Follow-1^b^ \| Follow-2^c^ \| \| --- \| --- \| --- \| --- \| --- \| \| 1 \|  \|  \|  \|  \| \| 2 \|  \|  \|  \|  \| \| 3 \|  \|  \|  \|  \| \| 4 \|  \|  \|  \|  \| \| 5 \|  \|  \|  \|  \| \| 6 \|  \|  \|  \|  \| \| 7 \|  \|  \|  \|  \| \| 8 \|  \|  \|  \|  \| \| 9 \|  \|  \|  \|  \| \| 10 \|  \|  \|  \|  \| \| 11 \|  \|  \|  \|  \| \| 12 \|  \|  \|  \|  \| \| 13 \|  \|  \|  \|  \| \| 14 \|  \|  \|  \|  \| \| 15 \|  \|  \|  \|  \| \| 16 \|  \|  \|  \|  \| \| 17 \|  \|  \|  \|  \| \| 18 \|  \|  \|  \|  \| \| 19 \|  \|  \|  \|  \| \| 20 \|  \|  \|  \|  \| \| 21 \|  \|  \|  \|  \| \| 22 \|  \|  \|  \|  \| \| 23 \|  \|  \|  \|  \| \| 24 \|  \|  \|  \|  \| \| 25 \|  \|  \|  \|  \| \| 26 \|  \|  \|  \|  \| \| 27 \|  \|  \|  \|  \|   ^a^ Seven days after operation.  ^b^ One month after operation.  ^c^ Three months after operation.  Table S2. Sequencing panel used in targeted capture hybridization. | | | | |
| --- | --- | --- | --- | --- | --- | --- | --- | --- | --- | --- | --- | --- | --- | --- | --- | --- | --- | --- | --- | --- | --- | --- | --- | --- | --- | --- | --- | --- | --- | --- | --- | --- | --- | --- | --- | --- | --- | --- | --- | --- | --- | --- | --- | --- | --- | --- | --- | --- | --- | --- | --- | --- | --- | --- | --- | --- | --- | --- | --- | --- | --- | --- | --- | --- | --- | --- | --- | --- | --- | --- | --- | --- | --- | --- | --- | --- | --- | --- | --- | --- | --- | --- | --- | --- | --- | --- | --- | --- | --- | --- | --- | --- | --- | --- | --- | --- | --- | --- | --- | --- | --- | --- | --- | --- | --- | --- | --- | --- | --- | --- | --- | --- | --- | --- | --- | --- | --- | --- | --- | --- | --- | --- | --- | --- | --- | --- | --- | --- | --- | --- | --- | --- | --- | --- | --- | --- | --- | --- | --- | --- | --- | --- | --- | --- |
| ABL1 | CDK4 | FGFR4 | MDM4 | PSMB1 |
| ABL2 | CDK6 | FLCN | MED12 | PSMB5 |
| AKT1 | CDK8 | FLT1 | MET | PTCH1 |
| AKT2 | CDKN1A | FLT3 | MITF | PTCH2 |
| AKT3 | CDKN1B | FLT4 | MLH1 | PTEN |
| ALK | CDKN2A | FOXA1 | MLH3 | PTPN11 |
| APC | CDKN2B | FOXL2 | MPL | RAF1 |
| AR | CHEK1 | GAB2 | MS4A1 | RARA |
| ARAF | CHEK2 | GATA3 | MSH2 | RB1 |
| ATM | CRKL | GNA11 | MSH3 | RET |
| ATR | CSF1R | GNAQ | MSH6 | RHEB |
| AURKA | CTNNB1 | GNAS | MTOR | RHOA |
| AURKB | DDR1 | HDAC1 | MYC | RICTOR |
| AXL | DDR2 | HDAC4 | MYD88 | RNF43 |
| BAP1 | DNMT3A | HGF | NF1 | ROCK1 |
| BCL2 | EGFR | HRAS | NF2 | ROS1 |
| BRAF | EPHA2 | IDH1 | NOTCH1 | RPS6KB1 |
| BRCA1 | EPHA3 | IDH2 | NOTCH2 | SMARCA4 |
| BRCA2 | EPHA5 | IGF1R | NOTCH3 | SMARCB1 |
| BRD2 | ERBB2 | IL7R | NOTCH4 | SMO |
| BRD3 | ERBB3 | INPP4B | NRAS | SRC |
| BRD4 | ERBB4 | IRS2 | NTRK1 | STAT1 |
| BTK | ERCC1 | JAK1 | NTRK3 | STAT3 |
| C11orf30 | ERG | JAK2 | PALB2 | STK11 |
| C1QA | ESR1 | JAK3 | PDGFRA | SYK |
| C1S | EZH2 | KDR | PDGFRB | TMPRSS2 |
| CBL | FAT1 | KIT | PDK1 | TOP1 |
| CCND1 | FBXW7 | KRAS | PIK3CA | TP53 |
| CCND2 | FCGR2A | MAP2K1 | PIK3CB | TSC1 |
| CCND3 | FCGR2B | MAP2K2 | PIK3R1 | TSC2 |
| CCNE1 | FCGR3A | MAPK1 | PIK3R2 | VEGFA |
| CD274 | FGFR1 | MAPK3 | PMS1 | VHL |
| CDH1 | FGFR2 | MCL1 | PMS2 | XPO1 |
| CDK13 | FGFR3 | MDM2 | PRKAA1 | XRCC1 |
| ABCA10 | ABCA8 | ABCB7 | ABCC8 | ABCF2 |
| ACE | ACER2 | ACOT11 | ACPP | ACSL1 |
| ACSM5 | ACSS3 | ACTL6B | ADAM23 | ADAM33 |
| ADAMTS12 | ADAMTS16 | ADAMTS19 | ADAMTS20 | ADAMTS5 |
| ADAMTSL1 | ADD2 | AGMAT | AGTPBP1 | AHCTF1 |
| AK5 | AKR1B10 | AKR1C1 | ALDH1A3 | ALDH2 |
| ALG5 | ALX4 | AMOT | ANK2 | ANKRD13D |
| ANKRD20A4 | ANKRD27 | ANKRD28 | ANKRD30A | ANKRD30B |
| ANKRD36B | ANO2 | AP1B1 | AP1G2 | AP3B1 |
| APAF1 | APLP2 | APMAP | APPL2 | AQP12A |
| ARFGAP1 | ARFRP1 | ARHGAP35 | ARHGAP40 | ARHGEF1 |
| ARHGEF7 | ARNTL | ARPC4-TTLL3 | ASH2L | ASTN1 |
| ASXL2 | ATAD2B | ATG9B | ATP10B | ATP10D |
| ATP12A | ATP2C1 | ATP6V0A2 | ATP8B2 | ATXN2 |
| ATXN7L2 | BAX | BBS9 | BCAS1 | BCAS2 |
| BLOC1S1 | BMPR1B | BRF1 | BRSK2 | BRWD3 |
| BSG | BTNL3 | BTRC | C12orf5 | C19orf38 |
| C1orf112 | C1orf35 | C20orf112 | C2orf47 | C2orf62 |
| C7orf53 | C9orf114 | C9orf43 | CACNA1A | CACNA1D |
| CACNA1E | CADM2 | CAMKK1 | CAPRIN1 | CARS |
| CARS2 | CASC4 | CASP8 | CASP8AP2 | CASQ2 |
| CATSPER2 | CBFB | CBX4 | CCDC155 | CCDC159 |
| CCDC17 | CCT3 | CCT6B | CD1E | CD300LF |
| CD5L | CD9 | CD97 | CD99 | CDH18 |
| CDH24 | CDH26 | CDK11A | CDK12 | CDK14 |
| CDK18 | CDK19 | CDS1 | CEACAM20 | CECR2 |
| CELA2B | CGN | CHD3 | CHD4 | CHD6 |
| CHI3L1 | CISD3 | CLCN7 | CLEC16A | CLINT1 |
| CNGB3 | CNKSR2 | CNOT3 | CNOT4 | CNTN1 |
| CNTN4 | CNTN5 | CNTNAP3B | CNTNAP5 | COASY |
| COL14A1 | COL16A1 | COL19A1 | COL1A1 | COL25A1 |
| COL4A5 | COL4A6 | COL5A1 | COL5A2 | COL5A3 |
| COL6A5 | COL6A6 | COL9A1 | COPA | COPG1 |
| CPA1 | CPSF3 | CPSF6 | CRTAM | CRTAP |
| CRYBG3 | CSMD1 | CSMD3 | CSN3 | CSNK1E |
| CSPP1 | CTCF | CTIF | CTNNA2 | CTSF |
| CYP2A13 | CYP3A4 | CYP4A11 | CYTH4 | DCLK2 |
| DCST1 | DDB1 | DDX24 | DDX3X | DEPDC4 |
| DGKK | DHCR24 | DHDDS | DHX9 | DIAPH1 |
| DKC1 | DLST | DMD | DMXL1 | DMXL2 |
| DNAH10 | DNAH5 | DNAH9 | DNAJC11 | DNAJC9 |
| DNTTIP1 | DOCK11 | DOCK3 | DOT1L | DPP10 |
| DPP4 | DRGX | DUOX1 | DYSF | DZANK1 |
| ECHDC1 | EDN1 | EEF1A1 | EFCAB5 | EFCAB6 |
| EFCAB7 | EFHA2 | EFNA5 | EIF1AX | EIF2B5 |
| EIF2C2 | EIF3E | EIF3I | EIF4ENIF1 | EIF4H |
| ELAVL3 | ELL3 | EMID2 | ENPP2 | ENTPD6 |
| EPB41L2 | EPB41L4B | EPHB1 | EPS8L3 | ESD |
| ETNK2 | ETV6 | EXOC4 | EXOC5 | EXOC6 |
| EXOC7 | EXTL3 | EYA4 | F8 | F9 |
| FAH | FAM114A2 | FAM131B | FAM135B | FAM13C |
| FAM157B | FAM177B | FAM21A | FAM3A | FAM49A |
| FAM49B | FAM5C | FAM86B1 | FAN1 | FANCC |
| FASTK | FATE1 | FBN2 | FDCSP | FLNC |
| FLOT2 | FLT3LG | FMN2 | FMNL3 | FNDC4 |
| FNIP2 | FOLH1 | FOXJ2 | FRG1 | FRG2B |
| FRMD4A | FRMPD2 | FRMPD4 | FSD2 | FSHR |
| FUBP1 | FUNDC1 | GAB3 | GABRD | GAD2 |
| GALNT13 | GALNT14 | GFRAL | GIGYF1 | GINS4 |
| GIPR | GKN2 | GLB1L3 | GLYR1 | GMDS |
| GNPTAB | GOLGA4 | GPAT2 | GPATCH2 | GPR114 |
| GPR125 | GPR133 | GPR144 | GPS2 | GRIA3 |
| GRIK2 | GUCY1A3 | GUCY2C | GYLTL1B | HAAO |
| HAP1 | HAUS5 | HAUS6 | HCN1 | HDAC6 |
| HEATR7B2 | HECTD4 | HECW1 | HECW2 | HID1 |
| HIST1H3B | HLA-DRB1 | HLA-DRB5 | HMCN1 | HMHA1 |
| HNF4A | HOMER2 | HPS3 | HPS4 | HSPA12B |
| HSPD1 | HYDIN | IBSP | IFT172 | IGSF9 |
| IKBKAP | IKBKE | IL11RA | IL13RA2 | IL1RAPL1 |
| IL27RA | IMPG1 | INHBA | INPP5J | IQCA1 |
| ITFG2 | ITGA8 | ITGA9 | ITIH1 | ITLN2 |
| ITM2A | ITPKB | ITPR1 | KCNAB2 | KCNH6 |
| KCNQ2 | KDM4A | KDM6A | KEAP1 | KIAA0195 |
| KIAA0226 | KIAA0319 | KIAA0922 | KIAA1191 | KIAA1199 |
| KIAA1211L | KIF13A | KIF1B | KIF26B | KIFAP3 |
| KIFC1 | KIR2DL3 | KIR3DL3 | KLHL1 | KLHL14 |
| KLK1 | KMT2B | KMT2C | KRT2 | KRT9 |
| KRTAP5-5 | KTN1 | L3MBTL1 | LARP1 | LCN10 |
| LCT | LCTL | LETM1 | LGALS13 | LILRB3 |
| LILRB4 | LIPN | LMAN1L | LMBR1L | LPCAT4 |
| LPHN3 | LRBA | LRP1B | LRP2 | LRP4 |
| LRRC16B | LRRC2 | LRRC7 | LRRC72 | LRRD1 |
| LRRFIP2 | LRSAM1 | LTBP1 | LUC7L2 | LUZP4 |
| MAEL | MAGI1 | MAN2A1 | MAP2 | MAP2K4 |
| MAP3K1 | MAP4K1 | MAPKAPK3 | MAPRE3 | MAST1 |
| MBIP | MBTPS2 | MCF2L2 | MCOLN2 | MDGA2 |
| MDN1 | MED23 | MEFV | METTL14 | METTL5 |
| MGAM | MICALL1 | MID1 | MIER2 | MLL |
| MLPH | MORC1 | MORN1 | MRPL1 | MRPL24 |
| MRPS18B | MSI1 | MTA2 | MTM1 | MTR |
| MTTP | MUC5B | MUS81 | MYB | MYBPC2 |
| MYCBP2 | MYH15 | MYH2 | MYH4 | MYH8 |
| MYH9 | MYL5 | MYL6 | MYLK2 | MYO3A |
| MYOM1 | NACAD | NARF | NAT10 | NAV3 |
| NBPF1 | NBPF10 | NCF2 | NCKAP1 | NCOR1 |
| NCOR2 | NEK5 | NELL1 | NFE2L2 | NIPBL |
| NLGN3 | NLRC3 | NLRP4 | NMI | NOP2 |
| NOS1 | NOS2 | NRXN1 | NRXN2 | NT5C3L |
| NTM | NUDCD2 | NUP205 | NUP210 | NUTM1 |
| NWD1 | NXF1 | NXF5 | OBP2A | OBP2B |
| OCA2 | ODZ3 | OR2T4 | OR4A15 | OR4C6 |
| OR5L2 | OR6F1 | OSBPL10 | OTOA | OTOGL |
| OVCH1 | P4HB | PABPC4 | PACS2 | PAEP |
| PAGE1 | PARK2 | PARP4 | PCK2 | PCLO |
| PCNT | PCNXL2 | PCSK5 | PCYT1A | PDCD6 |
| PDE1C | PDE2A | PDE4DIP | PDIA5 | PDILT |
| PDRG1 | PEX6 | PGAP1 | PHACTR3 | PHF20L1 |
| PHYH | PI4KB | PIP4K2C | PIP5K1C | PIWIL1 |
| PKD1L2 | PKHD1 | PKLR | PLAC8 | PLCB4 |
| PLCZ1 | PLEC | PLK2 | PLOD3 | PLXNA1 |
| POLDIP2 | POLE | POLR2J | POLR3B | POLR3GL |
| POLRMT | POM121L12 | POTEG | PPA1 | PPDPF |
| PPEF1 | PPFIBP2 | PPIL2 | PPP1R17 | PPP4R4 |
| PQBP1 | PREB | PREX2 | PRKACA | PRKAG3 |
| PRKCD | PRKDC | PRKX | PRRX1 | PRSS1 |
| PRUNE | PSG2 | PSG5 | PSIP1 | PSMC4 |
| PSMC6 | PSTPIP1 | PTBP3 | PTCD3 | PTGES3L-AARSD1 |
| PTGS2 | PTPLAD1 | PTPN13 | PTPRA | PTPRD |
| PTPRM | PYHIN1 | QRICH2 | RAB1B | RAB3GAP2 |
| RAB6A | RAC2 | RALBP1 | RAPGEF2 | RARB |
| RASEF | RBM6 | RBMX | RCC1 | REC8 |
| REG1B | RELN | RERE | RFWD2 | RFX3 |
| RNF215 | RNF219 | RPL22 | RPL36A | RPS5 |
| RPS6KA1 | RPTOR | RPUSD4 | RREB1 | RRP7A |
| RUNDC3A | RUNX1 | RYR2 | RYR3 | SAFB2 |
| SAG | SAGE1 | SAMD8 | SCN10A | SCN3A |
| SCN7A | SCN9A | SDK2 | SEC14L4 | SEC24B |
| SEH1L | SELP | SEMA6A | SEPT12. | SERPINA7 |
| SETD1B | SETD2 | SF1 | SF3B1 | SF3B14 |
| SF3B3 | SGCZ | SGIP1 | SGK1 | SGPL1 |
| SH2D3A | SH3BGR | SH3PXD2A | SHISA4 | SI |
| SIDT2 | SIK3 | SIM1 | SIM2 | SLC13A3 |
| SLC17A6 | SLC17A8 | SLC25A1 | SLC25A30 | SLC26A3 |
| SLC2A2 | SLC30A5 | SLC35B2 | SLC35B4 | SLC38A4 |
| SLC38A5 | SLC43A1 | SLC45A1 | SLC4A10 | SLC4A4 |
| SLC5A1 | SLC6A5 | SLC8A1 | SLCO1B7 | SLCO5A1 |
| SMTN | SNTG1 | SORCS3 | SPAG16 | SPATA13 |
| SPG20 | SPINT1 | SPPL2A | SPPL3 | SPRED1 |
| SPTA1 | SRRT | SSBP3 | SSH2 | SSPO |
| ST18 | ST6GALNAC1 | STAG2 | STAT4 | STAT6 |
| STK11IP | STK31 | STX3 | SULT1A4 | SUPT5H |
| SUPT6H | SYCP2L | SYNE1 | SYNE2 | SYNJ2 |
| TAF1B | TAF6 | TARBP1 | TBC1D1 | TBC1D21 |
| TBC1D3 | TBC1D5 | TBL1X | TBP | TBX15 |
| TBX22 | TBX3 | TCF20 | TCF4 | TCP10 |
| TCP11 | TEK | TESC | TEX35 | TFDP1 |
| TGDS | TGM2 | TGM5 | THBS2 | THEM5 |
| THOC1 | THSD7A | THSD7B | TIMD4 | TIMM44 |
| TIMP3 | TJP3 | TLE1 | TLL1 | TMC2 |
| TMED8 | TMEM104 | TMEM120B | TMEM132D | TMEM145 |
| TMEM247 | TMEM80 | TMEM87A | TMTC4 | TMX3 |
| TNFAIP6 | TNFSF4 | TNN | TNNT1 | TNR |
| TNS3 | TP53BP1 | TPCN1 | TPH2 | TPMT |
| TPTE | TRIM33 | TRIM51 | TRIM58 | TRIML1 |
| TRIO | TRIP11 | TRMT112 | TRPC5 | TRUB1 |
| TSGA10 | TSKS | TSPAN12 | TSR2 | TTF2 |
| TTN | TUBA3C | TUBGCP4 | TUBGCP5 | TYK2 |
| TYRP1 | U2AF1 | U2AF2 | UBASH3A | UBE2Q1 |
| UBE4B | UCHL3 | UCK2 | UGT8 | ULK3 |
| UMOD | UNC13A | UNC13D | UNC5D | USP12 |
| USP34 | USP39 | USP45 | USP48 | VAV1 |
| VEZF1 | VILL | VIT | VPS13A | VPS33B |
| VSIG4 | WAS | WASL | WDR44 | WDR52 |
| WDR62 | WDR66 | WDR72 | WDTC1 | WLS |
| WSCD2 | WWP2 | XBP1 | XPO4 | XPO5 |
| ZAP70 | ZBTB8OS | ZC3H13 | ZC3H7B | ZDHHC11 |
| ZFC3H1 | ZFR | ZMYM4 | ZNF143 | ZNF350 |
| ZNF385A | ZNF414 | ZNF512B | ZNF541 | ZNF563 |
| ZNF614 | ZNF687 | ZNF705B | ZNF705G | ZNF711 |
| ZNF804B | ZSWIM8 |  |  |  |
